# Transcriptome profiling of regulatory T cells from children with transient hypogammaglobulinemia of infancy

**DOI:** 10.1093/cei/uxad116

**Published:** 2023-11-04

**Authors:** Magdalena Rutkowska-Zapała, Agnieszka Grabowska, Marzena Lenart, Anna Kluczewska, Anna Szaflarska, Krzysztof Kobylarz, Anna Pituch-Noworolska, Maciej Siedlar

**Affiliations:** Department of Clinical Immunology, Institute of Paediatrics, Jagiellonian University Medical College, Wielicka, Krakow, Poland; Department of Medical Genetics, Institute of Paediatrics, Jagiellonian University Medical College, Wielicka, Krakow, Poland; Department of Clinical Immunology, Institute of Paediatrics, Jagiellonian University Medical College, Wielicka, Krakow, Poland; Department of Clinical Immunology, Institute of Paediatrics, Jagiellonian University Medical College, Wielicka, Krakow, Poland; Department of Clinical Immunology, Institute of Paediatrics, Jagiellonian University Medical College, Wielicka, Krakow, Poland; Department of Anesthesiology and Intensive Care, Institute of Paediatrics, Jagiellonian University Medical College, Wielicka, Krakow, Poland; Department of Clinical Immunology, Institute of Paediatrics, Jagiellonian University Medical College, Wielicka, Krakow, Poland; Department of Clinical Immunology, Institute of Paediatrics, Jagiellonian University Medical College, Wielicka, Krakow, Poland

**Keywords:** transient hypogammaglobulinemia of infancy, regulatory T cells, microarray analysis, transcriptome profiling, long non-coding RNA, microRNA precursors

## Abstract

Transient hypogammaglobulinemia of infancy (THI) is one of the most common forms of hypogammaglobulinemia in the early childhood. THI is usually associated with chronic, recurrent bacterial and viral infections, life-threatening in some cases, yet its pathogenesis is still largely unknown. As our previous findings indicated the possible role of T_reg_ cells in the pathomechanism of THI, the aim of the current study was to investigate gene expression profile of T_reg_ cells isolated from THI patients. The transcriptome-wide gene profiling was performed using microarray technology on THI patients in two time-points: during (THI-1), and in resolution phase (THI-2) of hypogammaglobulinemia. As a result, a total of 1086 genes were differentially expressed in THI-1 patients, when compared to THI-2 as well as control group. Among them, 931 were up- and 155 downregulated, and part of them encodes genes important for T_reg_ lymphocyte biology and function, i.e. transcription factors/cofactors that regulate FOXP3 expression. Thus, we postulate that T_reg_ cells isolated from THI patients during hypogammaglobulinemia display enhanced suppressor transcriptome signature. T_reg_ expression profile of THI children after normalization of Ig levels largely resembles the results obtained in healthy control group, suggesting THI T_reg_ transcriptome seems to return to that observed in healthy children. Taken together, we suggest that THI pathomechanism is associated not only with transiently elevated T_reg_ cell numbers, but also with their enhanced regulatory/inhibitory functions. These findings expand our knowledge of human T_reg_ cells and may be useful for the future diagnosis or management of THI.

## Teaser

Transient hypogammaglobulinemia of infancy (THI) is characterized by transiently reduced serum immunoglobulin levels and recurrent infections, accompanied by transiently elevated T_reg_ numbers. Here, we show that T_reg_ cells collected during the phase of hypogammaglobulinemia display enhanced suppressor transcriptome signature when compared to the resolution phase of THI. This signature is manifested by upregulated gene expression of T_reg_ master functional regulators, transcription factors, and TGF-β-signaling pathway mediators.

## Graphical Abstract

**Graphical Abstract:**
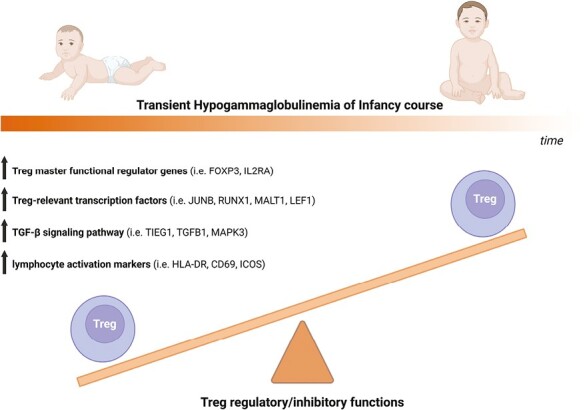

## Introduction

Human Inborn Errors of Immunity (IEI) also referred to as primary immunodeficiencies (PIDs) encompass, according to the latest update, 485 heterogeneous genetic diseases [1]. The most common forms of IEI, representing more than 50% of cases, are predominantly antibody deficiencies [1]. One of them, transient hypogammaglobulinemia of infancy (THI) is a heterogeneous disorder characterized by reduced serum IgG (and sometimes IgA) level in the early infancy, first described by Gitlin and Janeway in 1956 [2]. THI may be associated with recurrent infections, in some infants, but may remain asymptomatic in others [3, 4]. A putative diagnosis is initially made in children with IgG below age-related normal value detected in the first 3 years of life (measured at least twice), after exclusion of other causes of hypogammaglobulinemia. A definitive diagnosis of THI can only be established retrospectively in patients with spontaneous resolution before age 4 [5]. The underlying, definitive basis for this disorder may be heterogeneous and is still largely unknown.

In the last few years, the role of regulatory T cells (T_reg_) in immune dysfunction-based diseases has been intensively studied. T_reg_ cells are vital players in the maintenance of peripheral immunological self-tolerance and homeostasis, while the abnormalities (concerning number and/or functions) of these cells are observed in autoimmune and inflammatory diseases, allergic, infectious, and neoplastic disorders [6–9]. Moreover, latest reports consider the role of T_reg_ cells in the development and progression of different IEIs, such as common variable immunodeficiency (CVID) and Omenn syndrome [10, 11]. More importantly, we have previously shown a possible role of T_reg_ cells in the pathomechanism of THI [12]. In our study, the absolute number of circulating T_reg_ cells was significantly elevated in children with THI in comparison to healthy children and patients suffering from other primary humoral immunodeficiencies, such as selective IgA deficiency (SIgAD) or CVID. Prospective clinical observations performed in THI children revealed consistent decrease of T_reg_ numbers over time. The changes of T_reg_ number were also associated with normalization of IgG level and withdrawal of clinical symptoms, suggesting the association of the period of decreased IgG level in THI children with the transiently elevated absolute number of circulating T_reg_ lymphocytes [12]. It seems to be in line with previous observation that the suppressive effect of T_reg_ on B lymphocytes is also associated with decreased immunoglobulin production [13]. However, the causes of elevation of T_reg_ numbers in THI patients, or possible functional alterations of these cells are still unknown.

T_reg_ identity and function are determined by a unique T_reg_ “signature.” This “signature” comprises of a set of genes that are differentially expressed from conventional T cells [14, 15]. T_reg_ cells display suppressive functions, such as modulation of target cell signaling via cell–cell contact and/or secretion of immunosuppressive cytokines such as interleukin (IL) 10, IL-35 and transforming growth factor β (TGF-β) [16, 17]. Apart from coding transcriptome analysis, non-coding RNAs (ncRNAs) play an important role in post-transcriptional modulation of gene expression, which was shown to regulate lymphocyte functions [14, 18]. NcRNAs comprise of multiple classes of RNA transcripts that are not transcribed into proteins and play a significant role in gene regulation (including transcription, stability, or translation of protein-coding genes), both in health and disease [19]. NcRNAs, including microRNAs (miRNAs) and long non-coding RNAs (lncRNAs), regulate also important aspects of T_reg_ cells [20]. However, little is known about roles of ncRNAs in T_reg_ differentiation and their functions during the course of THI.

In this study, we aimed to determine the role of T_reg_ cells in the pathomechanism of THI, based on the gene expression profile analysis performed on isolated T_reg_ cells. As the study was performed in THI patients at two time points, during, and in resolution phase of hypogammaglobulinemia, we showed changes in the expression profile of genes important for T_reg_ lymphocyte biology and function. In parallel, we analyzed expression profiles of long intergenic non-coding RNA as well as microRNA precursors. This study improved our understanding of the role of T_reg_-related genes in the etiopathogenesis of THI.

## Materials and methods

### Patients

**Figure 1.**
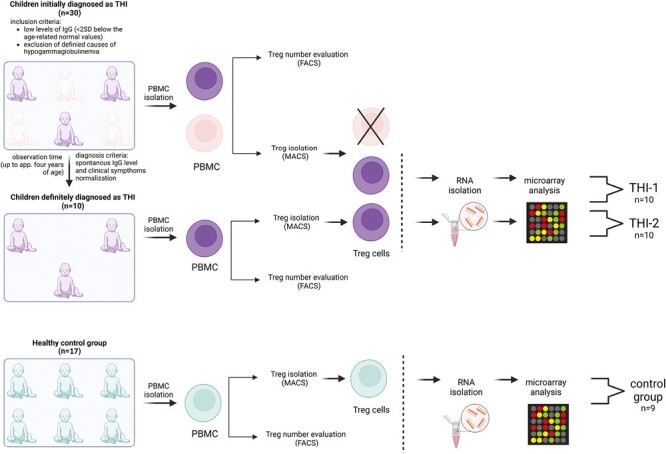
Study design. Two-step protocol was performed: (a) First T_reg_ cells number was assessed using flow cytometry (FACS) and next (b) T_regs_ were isolated using magnetic-activated cell sorting (MACS) technology and then frozen at −80°C until RNA isolation. The group of children definitely diagnosed as THI (*n* = 10), on the basis of diagnosis criteria, was formed from children initially diagnosed as THI (*n* = 30). RNA isolation followed by microarray analysis was performed in children with definitive THI diagnosis in two time-points: during hypogammaglobinemia (THI-1) and after Ig levels normalization (THI-2). Twenty-two healthy children were evaluated in this study in whom T_reg_ cells number was assessed. In nine of them, microarray analysis was performed.

We studied a cohort of 30 children who had been initially diagnosed with THI according to the following criteria: (1) low levels of IgG (<2 SD below the age-related normal values) detected in the first 3 years of life and (2) exclusion of defined causes of hypogammaglobulinaemia. At enrolment, none of the patients was affected by malignancies, treated with anti-neoplastic, or immunosuppressive drugs. Children born preterm were not included in the study. Only children without clinical symptoms of infection were enrolled in the study. Patients, whose IgG levels and clinical symptoms had spontaneously normalized before age 4, were diagnosed with definitive THI. Finally, 10 patients were definitively diagnosed as THI. T_reg_ cell numbers were evaluated in THI patients at two time points (during and after hypogammaglobulinemia), as well as in 17 age- and sex-matched healthy controls, whereas microarray analysis was performed in all 10 THI patients at two time points, during and after hypogammaglobulinemia, as well as in nine age-matched healthy controls. Twenty hypogammaglobulinemic infants who were suspected of THI but not diagnosed as THI in the end, formed “not THI” group. Detailed characteristics of all studied groups are shown in Table 1, while the scheme of the study is presented in Fig. 1. All patients were under supervision of the outpatient unit of the Department of Clinical Immunology of the University Children’s Hospital in Krakow. The study was approved by the Bioethical Committee of the Jagiellonian University (122.6120.2.2015 of 29 January 2015). A written informed consent was obtained from all the participants.

**Table 1.** Characteristic of patients and control group

Feature (*features of children in whom microarray analysis was performed)	Control	THI-1	THI-2
Number of children	17 (*9)	10	
Sex:F/M	8/9 (*3/6)	4/6	
Familial history of immunodeficiencies	0/17	0/10	
Autoimmune diseases	0/17 (*0/9)	0/10	
Allergy	0/17 (*0/9)	1/10	
Age (years ± SD),	2.63 ± 1.74 (*1.73 ± 1)	1.24 ± 0.56	3.33 ± 1.69
Age (in year and month) of particular THI patients:			
	Patient 1	2 years 3 months	5 years 3 months
	Patient 2	7 months	4 years 6 months
	Patient 3	1 years, 8 months	4 years 1 month
	Patient 4	1 year, 7 months	4 years 5 months
	Patient 5	1 year, 7 months	3 years 8 months
	Patient 6	10 months	2 years
	Patient 7	10 months	1 year, 10 months
	Patient 8	7 months	1 year 4 months
	Patient 9	11 months	2 years
	Patient 10	1 year, 7 months	4 years, 3 months

### T_reg_ cells number evaluation

Whole peripheral blood samples from all patients and healthy control subjects were drawn into EDTA-containing tubes (Vacutainer System; Becton Dickinson). For T_reg_ cells number evaluation, blood samples were incubated with anti-CD3-FITC (clone SK7) and anti-CD4-PE (clone SK3) (Becton Dickinson, San Jose, CA, USA) monoclonal antibodies (5 μL) in TruCount tubes (BD Biosciences), lysed and analyzed by flow cytometry using FACSCanto10 flow cytometer (Becton Dickinson Immunocytometry Systems, Palo Alto, CA) and analyzed with FACSDiva Software (BD Biosciences). The absolute numbers of CD3^+^CD4^+^ lymphocytes were calculated on a basis of bead and lymphocyte counts. For T_reg_ number evaluation, peripheral blood mononuclear cells (PBMC) were initially isolated by the standard Ficoll-Paque (Pharmacia Biotech) density gradient centrifugation. Then, isolated PBMCs were stained using the Human Regulatory T-cell Staining Kit (eBiosciences, San Diego, CA) consist of: mix of anti-CD4-FITC (clone RPA-T4) and anti-CD25-PE (clone BC96) mAbs (5 μL) and anti-Foxp3-APC (5 μL, clone PCH101). For all experiments, appropriate isotype-matched controls were included. The T_reg_ absolute numbers calculation was based on the absolute number of CD3^+^CD4^+^ lymphocytes.

### Isolation of regulatory T cells

T_reg_ cells were isolated from PBMCs. The isolation was performed in a two-step procedure using MACS technology and a Regulatory T Cell Isolation Kit II (Miltenyi Biotech), according to the manufacturer’s protocol. Briefly, cells were labeled with a cocktail of biotinylated antibodies and Anti-Biotin MicroBeads for the depletion of non-CD4^+^ and CD127^high^ cells. Then, the flow-through fraction of pre-enriched CD4^+^CD127^dim/–^ T cells was labeled with CD25 MicroBeads for subsequent positive selection of CD4^+^CD25^+^CD127^dim/–^ T_reg_ cells. LD and MS Columns (Miltenyi Biotech) were used during first (depletion) and second (positive selection) magnetic separation, respectively. After isolation, the cells were washed in MACS buffer, centrifuged for 10 minutes at 350 × *g*, suspended in 200 µL of RLT buffer (RNAeasy kit, Qiagen), mixed and kept frozen at −80°C until RNA isolation. Example data illustrating the purity of the isolated CD4^+^CD25^+^CD127^−/dim^ cells are shown in Supplementary Fig. S1A.

### Microarray analysis

Microarray expression analysis was performed for T_reg_ cell population isolated from THI patients (*n* = 10) and healthy control subjects (*n* = 9). The analysis of transcriptome-wide gene-level expression profiles was done with microarray technology using Clariom D Assays (Affymetrix). In brief, the total RNA was extracted from T_reg_ cells samples using RNeasy Micro Kit (Qiagen, Germany) according to the manufacturer’s protocol. DNA traces were removed from RNA samples by digestion with DNase I during the isolation process. The RNA concentration and quality were evaluated with NanoDrop 1000 Spectrophotometer (ThermoScientific). RNA Integrity Number (RIN) was analyzed using the TapeStation instrument and the High Sensitivity RNA ScreenTape System (Agilent Technologies). The obtained RINs ranged from 5 to 8.6. The input material used for microarray assays was 50 ng of total RNA. Samples for array hybridization were processed with GeneChip WT PLUS Reagent Kit and GeneChip 3000 instrument system (Affymetrix) in line of protocol. The Transcriptome Analysis Console (TAC) Software (Affymetrix) was used to analyze raw data for quality, expression patterns of genes, exons, lncRNA, and pathways. Gene advanced Robust Multiarray Analysis method with Signal Space Transformation (SST-RMA) summarization was performed by TAC (version 4.0.2.15 for Windows, Waltham, Massachusetts, USA, www.thermofisher.com). The quality of the experiment was assessed based on the values of Pos versus Neg AUC, intensity distribution plots, foreground-to-background plots, MA-plots, and 2D-intensity plots and principal component analysis (PCA). On the basis of the obtained data, outliers deviating from the others in the assumed parameters were excluded from further analysis. As we analyzed the same set of subjects in two time-point, repeated measure protocol was applied to remove the differences between individuals (inter-individual variability). The statistical analysis was performed with the Limma Bioconductor package (implemented in TAC 4.0.2) using linear modeling and empirical Bayes methods. Benjamini–Hochberg step-up FDR-controlling procedure was used in multiple hypothesis testing to correct for multiple comparisons. The microarray data have been submitted to the GEO database with accession number GSE226094 with the link https://www.ncbi.nlm.nih.gov/geo/query/acc.cgi?acc=GSE226094

### Quantitative real-time PCR (RT-qPCR) analysis

Microarray results were validated by RT-qPCR method using specific assays of primers and commercially available TaqMan probes for each gene target. Shortly, reverse transcription was performed using SuperScriptIII First-Strand Synthesis SuperMix (Invitrogen, ThermoFisher Scientific, USA). PCR reactions were performed in duplicates using TaqMan Gene Expression Master Mix (ThermoFisher Scientific, USA) and appropriate assays (ThermoFisher Scientific, USA): Eukaryotic 18S rRNA (Entrez gene ID: HSRRN18S, assay ID: Hs03003631_g1), FOXP3 (Entrez gene ID:50943, assay ID: Hs01085834_m1), IL2RA (Entrez gene ID: 3559, assay ID: Hs00166229_m1), CCR7 (Entrez gene ID: 1236, assay ID: Hs01013469_m1), and LEF1 (Entrez gene ID: 51176, assay ID: Hs01547250_m1). The RNA of the same patients who underwent microarray analysis was used for qPCR. Unfortunately, due to insufficient RNA amount left, qPCR was performed in only 7 of the 10 patients. Data were analyzed on QuantStudio 7 System (Applied Biosystems). The fluorescent signals generated during the informative log-linear phase were used to calculate the relative amount of mRNAs, while 18S was used as a control for each PCR run. The expression of each mRNA was calculated using 2^−ΔΔCT^ method.

### Statistical analysis

Statistical analysis was performed using GraphPad Prism version 8 (GraphPad Software Inc., San Diego, CA). Statistical differences between two groups were analyzed by Student *t*-test, Mann–Whitney test or Wilcoxon paired *t*-test where applicable. The normal distribution of values was verified using Shapiro–Wilk test. For multiple comparisons, non-parametric Kruskal–Wallis test with Dunn’s post hoc test was used. For parametric results, mean ± standard error of mean (SEM) was shown, while for nonparametric results—median ± interquartile range (IQR: Q1–25%, Q3–75%) was shown. The *P*-values < 0.05 were considered significant.

## Results

### T_reg_ cells number

**Figure 2.**
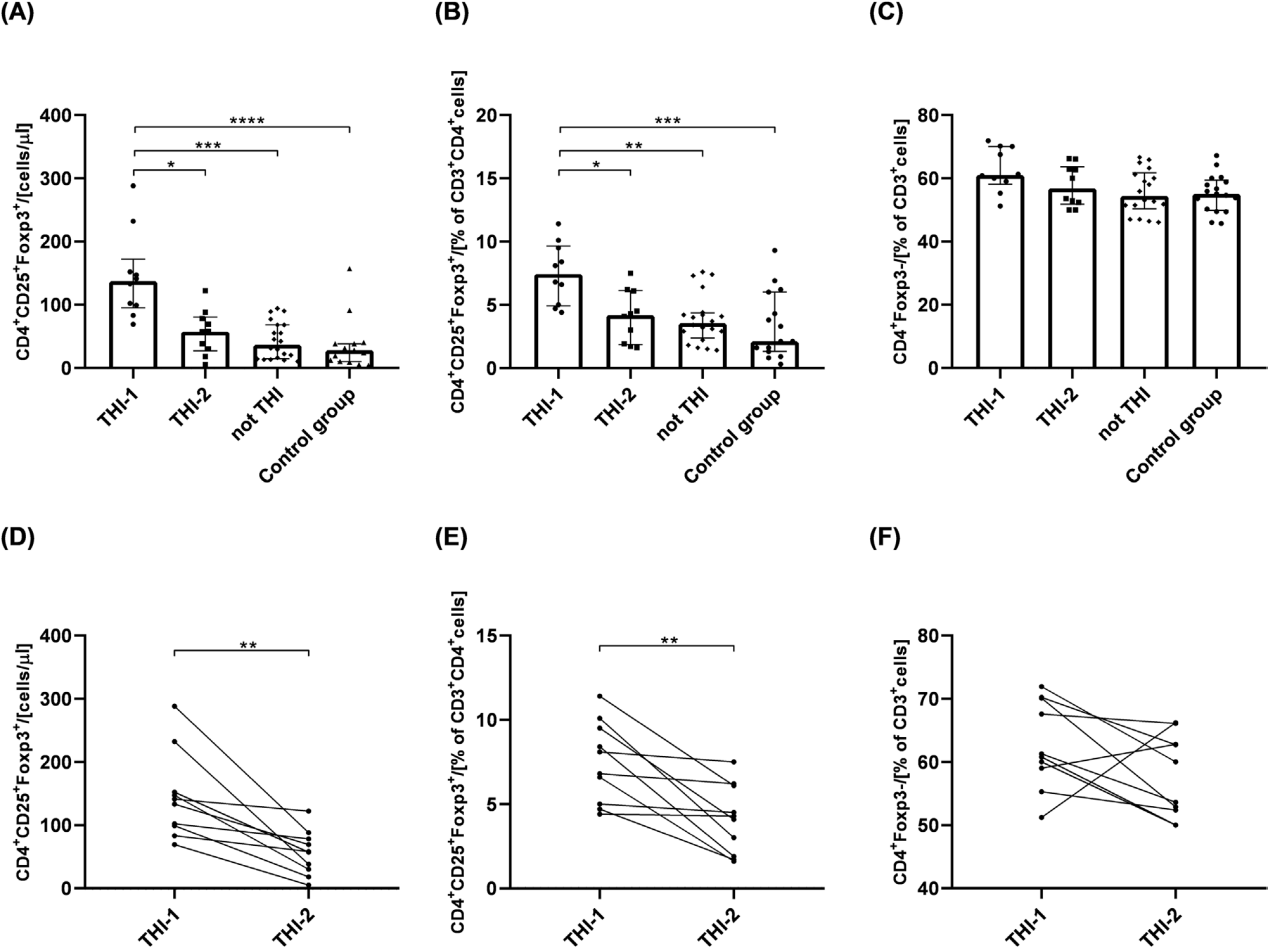
The absolute number of circulating T_reg_ cells and its percentage among CD3^+^CD4^+^ cells in all analyzed groups (a and b, respectively) and the changes of T_regs_ among the same THI patients in two time-points (d and e, respectively). The percentage of CD4^+^Foxp3^−^ cells in all analyzed groups (c) and their changes in THI patients in two time-points (f). The differences between studied groups were analyzed using Kruskal–Wallis (Figure a–c) or Wilcoxon paired *t*-test (Figure d–f). In the first case, median with interquartile range is shown. Asterisks mark significant differences: *P < 0.05, **P < 0.01, ***P < 0.001, ****P < 0.0001.

First, we analyzed the absolute numbers of circulating T_reg_ cells in children from THI-1 and THI-2 group and healthy controls, as well as “not THI” group. The latter consisted of hypogammaglobulinemic infants that were initially suspected of THI, yet, during the observation time, THI was excluded (Fig. 1). The gating strategy for T_reg_ cell FACS analysis is presented in Supplementary Fig. S1B. The results showed that THI patients during hypogammaglobulinemia (THI-1) had elevated numbers of T_regs_ compared to non-THI healthy subjects. However, following resolution of hypogammaglobulinemia (THI-2) T_reg_ levels returned to that seen in controls (Fig. 2a and b). Moreover, the proportion of circulating CD4^+^ non-T_regs_ in all groups was comparable (Fig. 2c). Individual changes in T_reg_ and non-T_reg_ cells for each THI patient at the two time points are shown in Fig. 2d–f. Also in this case, statistically significant changes were limited to T_reg_ cells (Fig. 2d and e).

### Gene expression pattern analysis in T_reg_ cells

Next, we performed the analysis of gene expression profile in T_reg_ cells isolated from THI patients during (THI-1) and after resolution of hypogammaglobulinemia (THI-2) as well as nine age-matched, to both THI groups, healthy controls, using microarray analysis. We used Clariom D assay, as a high-density array, which provides the most intricate transcriptome-wide gene level expression profile, including the ability to detect coding and long non-coding RNAs (lncRNAs). Filter criteria was fold change: >2 or <−2 and *P*-value < 0.05. Based on the positive versus negative AUC threshold, one sample from THI-1 group was detected as outlier; hence, excluded from further analysis together with corresponded sample from THI-2 group.

**Figure 3.**
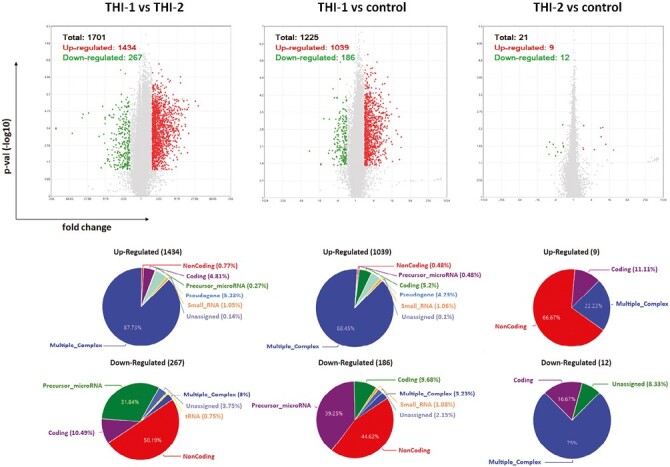
Results of microarray analysis of T_reg_ cells’ profile in all analyzed comparisons: THI-1 vs. THI-2, THI-1 vs. control, and THI-2 vs. control. Volcano plot showing differentially expressed transcripts (*P*-values < 0.05) with red spots representing upregulated and the green ones representing the downregulated genes. The grey colored region represents the non-significantly differentially expressed gene. The pie graphs, situated below each volcano plot, show upregulated (top) and downregulated (bottom) genes, according to their affiliation to one of nine groups of transcripts.

We performed analysis for all studied groups, that is, THI-1, THI-2, and healthy controls, comparing them in pairs. The graphical representation of detected differentially expressed genes (DEGs) in all analyzed comparisons, including volcano plots and pie graphs, are presented on Fig. 3. As a results, a total of 1701 genes were differentially expressed in THI-1 patients when compared to THI-2, which comprise about 1.25% of all analyzed genes. Among them, 1434 were upregulated and 267 downregulated. Most of the upregulated genes belonged to multiple complex group (87.73%), while the majority (50.19%) of downregulated genes were noncoding. Slightly less DEGs were detected when THI-1 group was compared with healthy controls. In that case, among 1225 DEGs, 1039 were up- and 186 were downregulated in THI-1 patients when compared to control group. The distribution of individual groups of DEGs was similar to the comparison of THI-1 with THI-2, that is, 88.45% of upregulated genes belonged to multiple complexes, whereas almost half of the downregulated genes were noncoding. Finally, the lowest number of DEGs was detected when T_reg_ cells from THI-2 and control group were compared. Here, among 21 DEGs, 9 were up-, while 12 were downregulated in THI-2 patients when compared to healthy controls. Among the upregulated genes, only three belonged to multiple complex or coding groups: CLC, GAPT, and HIF1A, while the rest six genes were noncoding. Twelve downregulated genes were: KIF2A, NAP1L1, NPIPB8, OST4, TC2N, SECISBP2L, RGS18, RSU1, PPBP, FCMR, and SAMHD1 and one noncoding.

**Figure 4.**
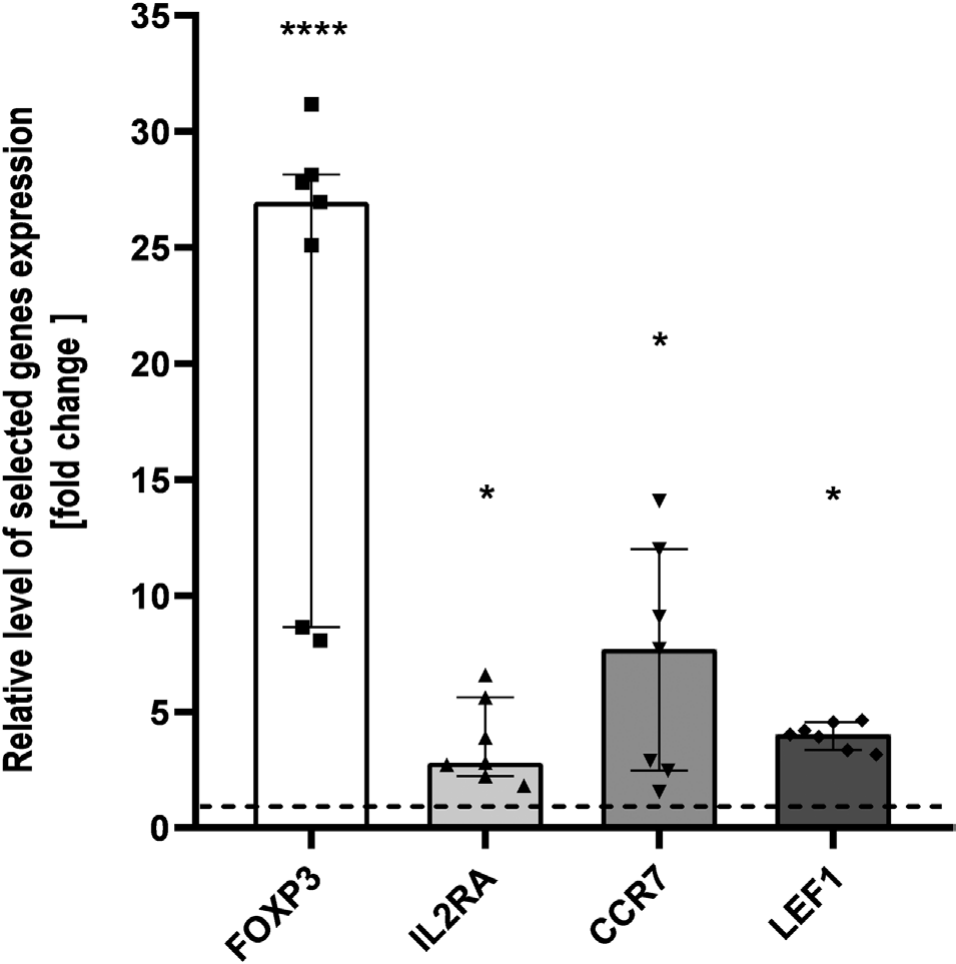
Microarray validation results. Relative expression of FOXP3, IL2RA, CCR7, and LEF1 validated by qPCR, presented as fold change of each DEG relative expression normalized to S18 expression and children from THI-2 group (2^−ΔΔCT^). The results were obtained from individual real-time PCR reactions performed in seven THI patients during (THI-1) and after resolution of hypogammaglobulinemia (THI-2). Dashed line was set on value 1 as it signifies the THI-2 group. Data were analyzed using non-parametric Kruskal–Wallis test with Dunn’s post hoc test. Median with interquartile range is shown. Asterisks mark significant differences: *P < 0.05, ****P < 0.0001.

To validate the microarray results, we performed real-time PCR reactions for four DEGs significant for T_regs_ biology and functions. The differences of FOXP3, IL2RA, LEF1, and CCR7 expression between THI-1 and THI-2 group reached statistical significance (Fig. 4) confirming microarray results. All mentioned genes were upregulated in THI patients during hypogammaglobulinemia (THI-1) when compared with these same patients with normal Ig levels (THI-2).

**Figure 5.**
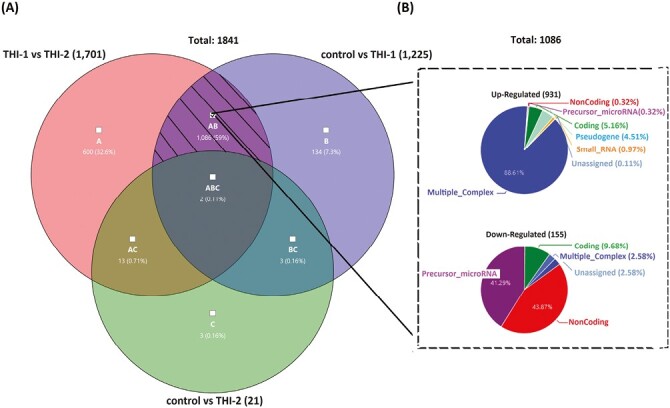
Venn’s diagram illustrating the overlap of the analyzed pairs of groups (a) and the pie graphs showing 1086 genes from the hatched field, divided into upregulated (top) and downregulated (bottom) genes, according to their affiliation to one of nine groups of transcripts.

**Figure 6.**
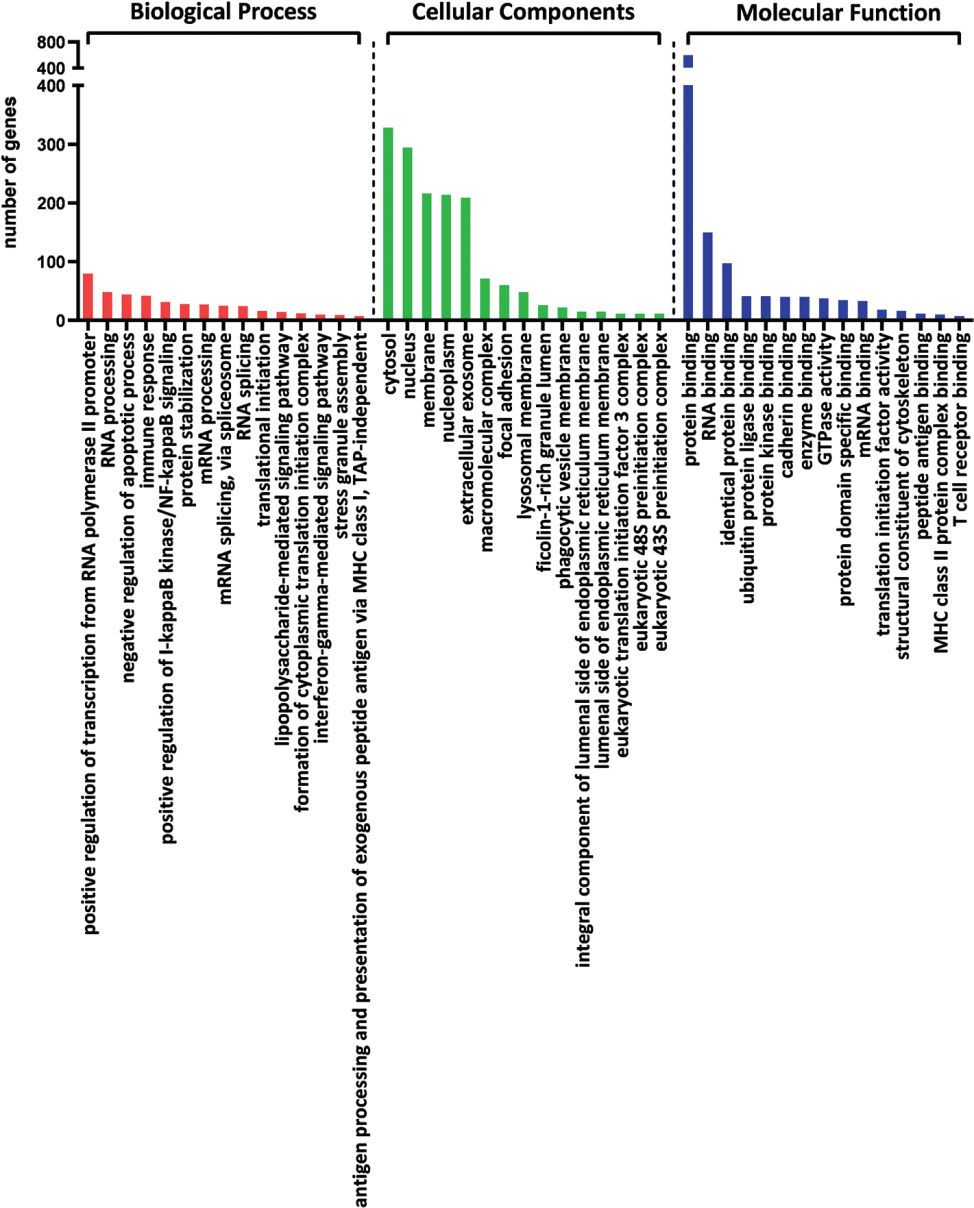
The bar chart depicts the top 15 Gene Ontology (GO) annotation categories including biological process, cellular component, and molecular function.

The common DEGs of all analyzed comparisons were evaluated by a Venn diagram (Fig. 5a). Our analysis focused on genes from the AB section (hatched field) which were differentially expressed in THI-1 group when compared to THI-2 as well as to healthy controls. Among 1086 genes from marked area, 931 were up- and 155 were downregulated (Fig. 5b). The majority of upregulated genes was classified as multiple complex, while almost half of downregulated DEGs belong to noncoding group. To investigate biological role of selected 1086 DEGs, enrichment analysis was performed using DAVID database [21]. The bar chart depicts the top 15 Gene Ontology (GO) annotation categories, including biological function, cellular component, and molecular function, is presented in Fig. 6. As a result, we observed that the key genes are enriched in biological process related to the positive regulation of transcription, RNA processing, and translation. In terms of cellular component, DEGs are associated mainly with cytosol, nucleus, cytoplasm, and membrane. In the molecular function category, the analyzed genes were mainly involved in protein, RNA, mRNA binding, or TCR receptor binding.

**Figure 7.**
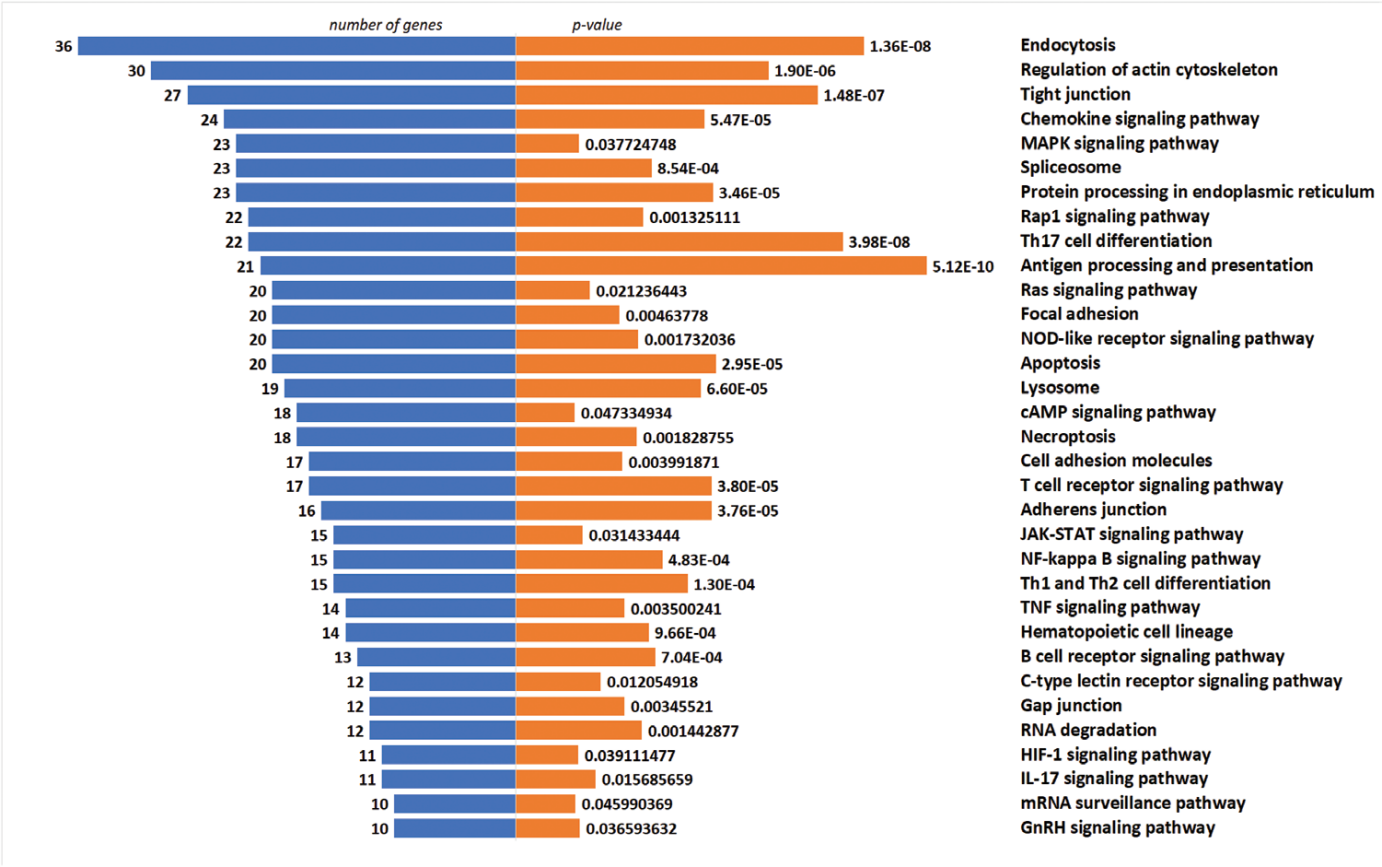
Top enriched KEGG pathways of DEGs, demonstrated by no. of genes and P-value.

To elucidate the cellular pathways associated with selected genes, KEGG pathway analysis was performed. The graphical presentation of the top pathway enrichment analysis is shown in Fig. 7. The analysis performed on 1086 genes showed that DEGs were primarily enriched in pathways strongly related to TCR signaling, such as antigen processing and presentation, T-cell receptor signaling pathway, but also related to Th17, Th1, and Th2 cell differentiation. The majority of pathways shown in Fig. 6 were previously described in the context of T_reg_ cells, such as the JAK3-STAT, Ras-PI3K, and TNF signaling pathways.

**Figure 8.**
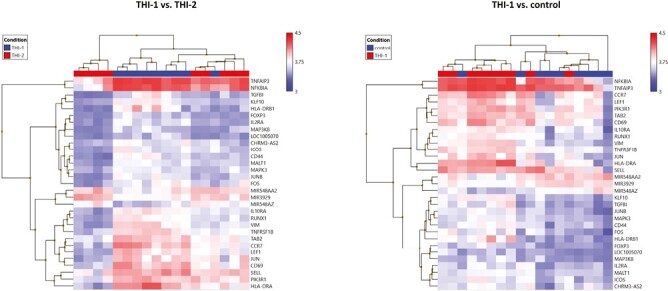
Heatmaps of core T_reg_ genes expression results. Color scale depicts minimum (blue) and maximum (red) normalized expression values compared across all samples between THI-1 and healthy controls (a) and THI-1 and THI-2 group (b).

Finally, we compared the core T_reg_ genes signature selected from 1086 genes and presented their expression on a heatmap in two pairs of comparisons: THI-1 versus THI-2 and THI-1 versus healthy controls (Fig. 8). The detailed characteristics of these genes, including their fold changes in the analyzed comparisons, are presented in Table 2. As a result, T_reg_ cells isolated from THI-1 patients, in comparison to healthy subjects as well as THI-2, expressed higher transcripts levels of T_reg_ marker genes including FOXP3 and IL2RA. However, we did not observe differences in expression of NRP-1, GITR, CD45RA, IKZF2, TIGIT, and CTLA4. As we observed increased FOXP3 expression level in THI-1 children in comparison to THI-2 and control group, in further analysis, we investigated which of the known transcription factors, interacting with FOXP3, were also differentially expressed. As a result, we found several DEGs previously described as epigenetic modifiers of FOXP3 expression, like: JUNB, MALT1, RUNX1, and LEF1. They all were upregulated in THI-1 patients in comparison to THI-2 and healthy controls. JUNB encodes TFs that regulate FOXP3 expression downstream of TCR signaling. MALT1 seems to play a central role in signaling cascade of CARD11-BCL10-MALT1 (CBM) that mediates TCR-induced NF-κB activation in T_reg_ cells and controls the conversion of T_reg_ cells from resting to effector stage under homeostatic conditions [22]. RUNX1 can regulate FOXP3 expression and its promoter activity [23, 24], while LEF1 act redundantly as FOXP3 cofactors to enhance its transcriptional activity [25]. Furthermore, as T_regs_ develop also in the periphery being induced by transforming growth factor-β (TGF-β) [16, 17], we analyzed expression level of genes related to this signaling pathway. From 1086 selected DEGs, 20 belong to this pathway, and they were all upregulated in THI-1 patients when compared both, with THI-2 and control group. Among them, upregulation of TIEG1 (KLF10), TGFBI, MAPK3, and MAP3K8 genes were observed. In our study, also VIM expression was higher in THI-1 patients when compared with THI-2 group as well as with healthy controls, with fold change 7.11 and 6.57, respectively.

**Table 2.** Characteristics of highly significant T_reg_ relevant DEGs obtained by comparing THI-1 with THI-2 and control group. Positive fold change value indicates an increase of expression, while a negative fold change indicates a decrease in expression between compared pairs

Gene Symbol/gene synonyms	Description	Group	Fold change for THI-1 when compared with THI-2 (*P*-value)	Fold change for THI-1 when compared with control [*P*-value)
ICOS	Inducible T-cell costimulator	Coding	3.08 (0.0003)	2.62 (0.0024)
HLA-DRA	Major histocompatibility complex, class II, DR alpha	Coding	15.72 (0.0003)	12.67 (0.0007)
FOXP3	Forkhead box P3	Coding	2.83 (4.25E-05)	2.51 (0.0008)
IL2RA	Interleukin 2 receptor, alpha	Coding	3 (7.76E-05)	2.55 (0.0053)
CCR7	Chemokine (C-C motif) receptor 7	Coding	10.34 (0.0001)	5.39 (0.003)
TNFRSF1B/TNFR2	Tumor necrosis factor receptor superfamily, member 1B	Multiple complex	4.16 (3.38E-05)	3.1 (0.0012)
JUN	Jun proto-oncogene	Multiple complex	8.55 (0.0006)	12.41 (5.94E-05)
SELL/CD62L	Selectin L	Multiple complex	2.72 (0.0181)	2.15 (0.0356)
LEF1	Lymphoid enhancer-binding factor 1	Multiple complex	5.99 (0.0003)	2.32 (0.0157)
PIK3R1	Phosphoinositide-3-kinase, regulatory subunit 1 (alpha)	Multiple complex	3.03 (0.0137)	2.78 (0.007)
TGFBI	Transforming growth factor, beta-induced, 68kDa	Multiple complex	2.85 (0.0048)	2.53 (0.029)
KLF10/TIEG	Kruppel-like factor 10	Multiple complex	4.69 (0.0007)	5.32 (0.0052)
TNFAIP3	Tumor necrosis factor, alpha-induced protein 3	Multiple complex	3.58 (0.0043)	2.16 (0.0148)
TAB2	TGF-beta activated kinase 1/MAP3K7 binding protein 2	Multiple complex	4.1 (0.0003)	2.08 (0.017)
HLA-DRB1	Major histocompatibility complex, class II, DR beta 1	Multiple complex	9.05 (0.0001)	6.23 (0.0003)
VIM	Vimentin	Multiple complex	7.11 (3.05E-05)	6.57 (7.01E-05)
MAP3K8	Mitogen-activated protein kinase kinase kinase 8	Multiple complex	2.13 (0.0079)	2.04 (0.0071)
CD44	CD44 molecule (Indian blood group)	Multiple complex	3.63 (0.0002)	2.26 (0.01)
IL10RA	Interleukin 10 receptor, alpha	Multiple complex	3.96 (0.0002)	2.34 (0.0036)
CD69	CD69 molecule	Multiple complex	8.8 (3.41E-05)	5.86 (6.29E-05)
FOS	FBJ murine osteosarcoma viral oncogene homolog	Multiple complex	2.76 (0.0179)	4.75 (0.0007)
NFKBIA	Nuclear factor of kappa light polypeptide gene enhancer in B-cells inhibitor, alpha	Multiple complex	7.86 (0.0091)	7.14 (0.0052)
MAPK3	Mitogen-activated protein kinase 3	Multiple complex	2.37 (0.0058)	2.08 (0.0016)
MALT1	MALT1 paracaspase	Multiple complex	2.81 (0.0001)	2.1 (0.004)
JUNB	Jun B proto-oncogene	Multiple complex	2.78 (0.0003)	2.35 (0.0003)
RUNX1	Runt-related transcription factor 1	Multiple complex	3.97 (0.0001)	2.35 (0.0039)
LOC100507006	Antisense to PELI1	Non-coding	2.1 (0.0002)	2.13 [*0.0002*]
CHRM3-AS2	CHRM3 antisense RNA 2	Non-coding	2.92 (0.0006)	2.53 [*0.0043*]
MIR548AZ	MicroRNA 548az	Precursor microRNA	−2.71 (0.0012)	−2.3 (0.0029)
MIR548AA2	MicroRNA 548aa-2	Precursor microRNA	−2.19 (0.0016)	−2.47 (0.0024)
MIR3929	MicroRNA 3929	Precursor microRNA	−3.73 (4.93E-05)	−2.29 (0.0231)

As the non-coding RNAs accounted for a significant percentage of 1086 DEGs selected using Venn diagram, we delved their expression more. Among 71 selected DEGs classified as non-coding, only two were described previously, that is, transcripts antisense to CHRM3 (fold change 2.53 and 2.92 in THI-1 when compared with control and THI-2, respectively) and antisense to PELI1 (fold change 2.13 and 2.1 in THI-1 when compared with control and THI-2, respectively). In our study, we did not observe differences in the expression of CHRM3 or PELI1 which the antisense transcripts overlap. Another fraction of differentially regulated transcripts were transcripts classified as (miRNA) precursors. Here, we found 67 genes which expression varied between THI-1 when compared to THI-2 patients and healthy controls, including MIR548AA2, MIR3929, and MIR548AZ.

## Discussion

T_reg_ cells play a role in the pathogenesis of several immune-based diseases, including immunodeficiencies [7–12]. We have proven, here and previously [12], that the course of THI disease is associated with transiently elevated T_reg_ cell numbers. Based on these findings, we suggested that T_reg_ count may become a THI-specific predictive biomarker.

Moreover, we show that not only the number of T_reg_ cells but also their transcriptional alterations may be related to the pathogenesis of THI. DEGs detected in our study appear to be involved in T_reg_ development and function-causing pathways. For example, JAK3-STAT signaling pathway is involved in the initiation and maintenance of Foxp3 in T_regs_ and has been associated with demethylation of the intronic Conserved Non Coding Sequence-2 (CNS2) [26], while Ras–PI3K pathway could modulate T_regs_, as PI3K/AKT signals are necessary for their development and function [27]. Another dysregulated pathway identified in our studies was associated with TNF signaling. It has been shown to be important for controlling T_reg_ homeostasis, differentiation, and proliferation [28]. What is more, one of the 14 genes differentially expressed in TNF pathway and upregulated in THI-1 patients, was tumor necrosis factor receptor 2 (TNFR2, TNFRSF1B). Recent studies have shown that TNFR2 is preferentially expressed by T_regs_ and that the expression of this receptor identifies maximally suppressive T_regs_. Moreover, TNF activates and expands T_regs_ through TNFR2 and recent studies shown that NF-κB, MAPK, and PI3K-Akt pathways are required for the inhibitory effect of T_regs_. First two, NF-κb and MAPK pathways, are also dysregulated in our study.

TGF-β signaling in peripheral T_regs_ seems to be essential for the regulation of peripheral T_reg_ numbers and for their immunosuppressive function *in vivo* [29]. We did not observed differences in expression level of genes encoding receptors for TGF-β, i.e. TβRI and TβRII, and this is consistent with our previous findings, showing that THI is not associated with mutations or polymorphisms in TGF-receptor coding genes [30]. We might, although, suggest that T_reg_ functional alterations in THI is rather associated with TGF-β signaling pathways than the receptors alone.

Finally, none of the 138 DEGs classified as ncRNAs have been described previously in the context of T_reg_ cells, except for one, that is, antisense to PELI1, which was upregulated in children with THI during hypogammaglobulinemia. Overexpression of PELI1 gene promotes proliferation and enhances the immunosuppressive function of T_reg_ cells *in vitro* [31]. Although we did not observe dysregulation of PELI1 in our study, it is possible that it is related to T_reg_ cells. Thus, we cannot exclude that our observation regarding increased expression of antisense to PELI1 might be clinically relevant for THI studies in the future; however, this issue requires further study. Finally, since both miRNA precursors and non-coding RNAs are essential gene expression regulators in eukaryotes, we suppose that at least several of these transcripts detected as differentially expressed, may have a potential effect on T_reg_ cells [32]. However, the functions of most of these transcripts remain unknown.

Our results strongly suggest that THI is associated with T_regs_ enhanced regulatory (inhibitory) functions. A number of DEGs upregulated in THI-1 support the hypothesis of their more activated state. As an example, LEF1 is required for the optimal response to IL-2 in T_reg_ cells and the repression of non-T_reg_ lineage genes [33]. Moreover, it is likely that LEF1 can antagonize the effects of IRF4 in T_reg_ homeostatic differentiation [34]. This hypothesis seems to be confirmed by our results, as the expression level of IRF4 do not differ between the groups. However, in a parallel study, Wang and Fu found that the expression of LEF1 in T_reg_ cells seemed to decrease with the transition of these cells from the resting, through activated, to the effector phase [34]. VIM, encodes vimentin, a cytoskeletal protein, which is critical not only for maintaining structural integrity in circulating lymphocytes, but also seems to participate in the negative regulation of T_reg_-cell functions [35]. Vimentin promotes a signaling that restrains T_reg_ activity during immune responses, without altering the T_reg_ survival or homing. What is more, T_reg_ cells from THI-1 children had increased expression of lymphocyte activation markers, such as CD69, HLA-DR, or ICOS [36–38], suggesting more activated status of these cells.

T_reg_ cells have been shown to be greatly heterogonous population. They can be divided on the basis of their origin (thymus-derived vs. peripheral), suppressor activity status (resting/naïve vs. effector), or even their target (i.e. follicular T_regs_). Each subset might be characterized by different surface antigen expression or intracellular protein expression [39–41]. In our study, we observed differential expression of T_reg_ subsets marker such as FOXP3, CD62L, CD44, and CCR7, which were upregulated in the THI-1 group. Nonetheless, we cannot strictly determine the T_reg_ subpopulation that prevail in THI-1 children. FOXP3, in combination with CD45RA, has been proposed as the best marker for the division of human T_reg_ cells into two populations: resting (CD45RA + FOXP3low) and activated (CD45RA − FOXP3high) [42]. Other T_reg_ classification is based on CD62L and CD44 expression and divides T_reg_ cells into CD62L^+^CD44^low^ cells, which seem to be resting (or naïve) and CD62L^−^CD44^hi^ that seem to comprise the activated (or effector) pool of T_regs_ [25, 43, 44]. CD44 was described as T_regs_ marker in mouse studies [45]. Upregulated CCR7 expression level could reflect more “resting” or “naïve” character of these cells, in contradiction to “activated” or “effector” T_reg_ cells, which show enhanced migration through non-lymphoid tissues [25, 43, 44]. Moreover, we did not detect differential expression of chemokine receptor CXCR5, characteristic for follicular T_reg_ (Tfr) cells, which are specialized in suppression of humoral responses, regulating antibody production following antigen exposure [46, 47]. Nonetheless, microarray analysis results do not reflect the complexity of cellular lineage subpopulations and for their studies other laboratory techniques are required.

Finally, the T_reg_ transcriptome signature seems to return to that observed in healthy children, as there was low number of DEGs between the THI-2 and control group. These observations support our suggestion of dysregulated T_reg_ functions and their higher activation in THI, assuming their probable role in the pathomechanism of this immunodeficiency. On the other hand, in THI-2 children, we observed altered expression of i.e. genes encoding modulators of T_reg_ development, such as SAMHD1 and HIF1A that degrade Foxp3 [48, 49], suggesting long-term alteration of T_regs_ accompanying this disease. Nevertheless, the underlying direct cause of the increased number and disturbed function of Tregs in children with THI remains to be determined.

Alternatively, we may also hypothesize that highly activated T_regs_ state observed in THI-1 children is essential in the resolution of THI. T_regs_ might expand rapidly, become highly activated, expressing all core functional T_reg_ genes. As a result, in the resolution phase of THI, increased T_regs_ activity leads to contraction of immune response, resulting in normalization of immunoglobulin production. However, to support this hypothesis, additional studies are needed, especially of Tfh and Tfr cells.

There are some limitations of our study. First, microarray analysis was performed in low-number groups of patients with THI and healthy controls. The number of children assigned to the THI group was low because of the low prevalence of this type of immunodeficiency and its problematic final diagnosis, which requires long-term observation and can only be made retrospectively. However, we want to emphasize that our patient group was a unique cohort of the same infants in which T_regs_ were analyzed during and after disease resolution. Regarding the control group, it was difficult to collect a higher number of healthy children because of the age limitation that would correspond to the mean age of the THI groups among the children admitted to our Outpatient Clinical Immunology Unit. Second, the microarray analysis results would be greatly supplemented by T_reg_ functional analysis. Here, we were unable to perform any additional assays due to very small amount of blood that could be collected from the patients, limited by their age, bioethical restrictions, and the amount of RNA required for microarray analysis. Third, gene profile analysis was limited to genes with known or possible associations with T_reg_ functions. We cannot exclude the possibility that analysis of the expression of other genes in these cells would also be clinically relevant.

In summary, we suggest that THI pathomechanism is associated not only with transiently elevated T_reg_ cells numbers, but also with their enhanced regulatory (inhibitory) functions. T_reg_ cells of THI-1 patients display enhanced suppressor transcriptome signature, most importantly consisting of T_reg_ master functional regulators and TGF-β signaling pathway mediators. These findings expand our knowledge of human T_reg_ cells and may be useful for the future diagnosis or management of THI.

## Supplementary Material

uxad116_suppl_Supplementary_Figures_1

## Data Availability

The microarray data have been submitted to the GEO database with accession number GSE226094 with the link https://www.ncbi.nlm.nih.gov/geo/query/acc.cgi?acc=GSE226094

## Abbreviations:

DEG: differentially expressed gene
THI: Transient Hypogammaglobulinemia of Infancy
T_reg_: regulatory T cell
