# Supplementary figures and images for: Transcriptome profiling of regulatory T cells from children with transient hypogammaglobulinemia of infancy
Source: Clin Exp Immunol. 2023 Nov 4;214(3):275–88. doi: 10.1093/cei/uxad116 (PMC10719223; doi:10.1093/cei/uxad116)

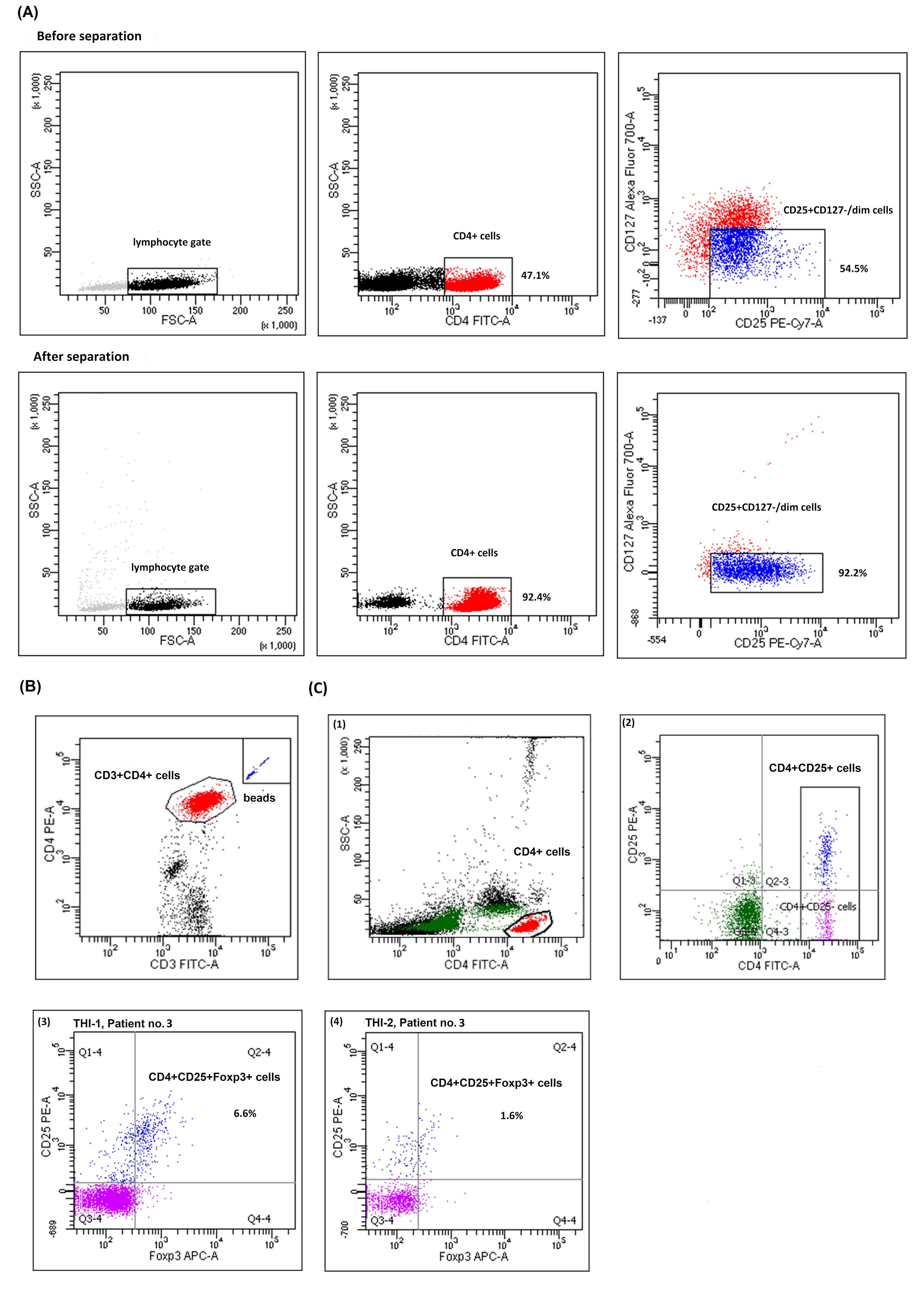

Supplement: uxad116_suppl_Supplementary_Figures_1 [file uxad116_suppl_supplementary_figures_1.jpeg]
